# Molecular Evaluation of Kyoho Grape Leaf and Berry Characteristics Influenced by Different NPK Fertilizers

**DOI:** 10.3390/plants10081578

**Published:** 2021-07-30

**Authors:** Muhammad Fiaz, Chen Wang, Muhammad Zia Ul Haq, Muhammad Salman Haider, Ting Zheng, Ge Mengqing, Haifeng Jia, Songtao Jiu, Jinggui Fang

**Affiliations:** 1 Key Laboratory of Genetics and Fruit Development, College of Horticulture, Nanjing Agricultural University, Nanjing 210095, China or fiaz.m2002@gmail.com (M.F.); wangchen@njau.edu.cn (C.W.); salman.hort1@gmail.com (M.S.H.); 2015204002@njau.edu.cn (T.Z.); 2019204016@njau.edu.cn (G.M.); jiahaifeng@njau.edu.cn (H.J.); 2 Department of Agronomy, University of Agriculture, Faisalabad 38000, Pakistan; ziaagr@yahoo.com; 3 Department of Plant Science, School of Agriculture and Biology, Shanghai Jiao Tong University, Shanghai 200240, China; jiusongtao@sjtu.edu.cn

**Keywords:** Kyoho grapes (*V. vinifera* × *V. labrusca*), commercial fertilizers, anthocyanin profile, nitrogen pathway genes, MYB pathway genes

## Abstract

Fertilization, a fundamental aspect of a plant’s life, has been of great concern for agricultural specialists to minimize the yield gap between actual and potential yield. Around the globe, fertilizers with different NPK ratios are being used to attain a better yield of grape. To find the suitable commercially available fertilizer for quality grape production, a 2 years (2017–2018) study was conducted for the evaluation of 10 fertilizers with different NPK ratios. Commercial fertilizers included were Zhanlan (16:16:16), Garsoni (15:15:15), Acron (16:16:16), Norway (21:7:12), Peters 1 (30:10:10), Nutrivant (14:14:30), Peters 2 (20:20:20), UMAX (15:15:15), G2 (20:20:20), and Yara (15:15:15). The fertilizer application rate was 20 g plant^−1^, and each was applied at L-29, L-33, and L-36 phenological stages. Chlorophylls, carotenoids, macro/micronutrients in leaf, and anthocyanin derivatives in grape peel were evaluated. Expression levels of 24 genes, including nitrogen, phosphorous, potassium, and anthocyanin pathways in leaf, peel, and pulp were validated by qPCR at L-29, L-33, and L-36 stages. Results indicated that Norway (21:7:12) and Peters 1 (30:10:10) increased carotenoids, chlorophylls, and anthocyanins in leaves, while Zhanlan (16:16:16) improved fruit biochemical attributes, and anthocyanin (cyanidin, delphinidin, petunidin, malvidin, peonidin, and pelargonidin contents). However, a better grape yield was obtained by the application of Peters 1 (30:10:10). Potassium pathway genes were upregulated by Nutrivant (14:14:30), phosphorous pathway genes by Peters 2 (20:20:20), and nitrogen pathway genes by Peters 1 (30:10:10), while Nutrivant (14:14:30) upregulated anthocyanin pathway genes and simultaneously enhanced anthocyanin biosynthesis in berry peels. Results of two years’ study concluded that Peters 1 (30:10:10) was proved better to increase yield, while Zhanlan (14:14:30) was superior in improving anthocyanin biosynthesis.

## 1. Introduction

Soil fertility decline is a major prevailing factor in reducing food production in tropical regions. Tumbling fertility is mainly attributed to the erosion of mineral via harvested crop, depletion of organic matters, and less addition of inorganic fertilizers [1]. Therefore, the application of fertilizer is increasing to maintain soil fertility. According to the Food and Agriculture Organization, the current world demand for nitrogen, potassium, and phosphorus fertilizers has reached 194.857 million tons in 2020, as it was 191.981 million tons in the previous year [2]. Grapevine is among the most economically important fruit crops [3] and exhibits quick response to fertilizers, being a heavy NPK feeder. One of the essential viticulture techniques is the fertilization of the soil that plays a significant role in both yield and grape quality. Nevertheless, unbalanced and excessive fertilizer, regardless of application time, causes not only yield shortage, but also impose negative impacts on quality components. Farmers apply fertilizers traditionally based on personal experience and environmental factors of specific regions that exhibit variation in type and dose of fertilizers [4,5]. Phenotypic and physiological processes depend on optimum nutrients availability, especially N, P, and K are macronutrients, and therefore are very essential for crops [6,7] to increase growth and yield potential of plants [8].

Increasing the amount of N applied during the period of early growth promotes vegetative growth, which results in high yields. However, excessive N fertilizer applications impede differentiation of flower buds and yield related attributes [9,10]. Increasing N within plants can increase young grapevines vigor growth [11]. This can also increase the number of clusters per grapevine. In addition, N increment thus decreases total anthocyanins (TA) [12], dilutes total soluble solids (TSS), and increases total titratable acidity in the must [13,14]. Wine-makers in traditional grape-growing countries are continuously seeking the perfect composition of the must and wine because of the strong positive correlation with the wine price. Therefore, focus is increasing on appropriate grapevine nutrition [12,13].

Potassium contributes a role in many functions in plants; it increases the photosynthesis rate, maintain turgor of stomata guard cell, balance osmotic concentrations, increase biomass production, and yields [14,15,16,17]. Similarly, potassium applications to grapevine increase berry acidity, sugar, and anthocyanin contents; additionally, they improve berry weight and firmness [13,14]. Phosphorous impacts root morphology and development, to increase water and nutrient absorption, which favors in effective drought mitigation and yield improvement [18,19,20,21]. The application of adequate NPK fertilizer to a vineyard renders a positive influence on the structural elements of plants, mechanical compositions, quality of grape berries, and yield [22]. The enhancement of anthocyanin contents was observed by the limited supply of NP fertilizers as deficiency of certain nutrients enhanced the transcription of genes involved in flavonoid pathway. Genetic makeup, cultivation conditions, and plant hormone levels control the production of anthocyanins.

In the assimilation process of nitrogen, major enzymes are encoded by the grapevine nitrogen metabolism genes, including *VvNR* (*V. vinifera* nitrate reductase), *VvGDH* (*V. vinifera* glutamate dehydrogenase), *VvAS* (*V. vinifera* asparagine synthetase), *VvNIR* (*V. vinifera* nitrite reductase), and *VvGS* (*V. vinifera* glutamine synthetase) [23]. Potassium and phosphorous is absorbed and transported by K and P channel genes like *VvSORK* (*V. vinifera* outward rectifying shaker-like K+ channel), *VvSIRK* (*V. vinifera* inward rectifying shaker-like K+ channel), *VvKUP2* (*V. vinifera* K+ uptake and metabolism permeases 2), *VvKUP1* (*V. vinifera* K+ uptake and metabolism permeases 1), *VvPAP* (*V. vinifera* purple acid phosphatase), *VvPHO1* (*V. vinifera* phosphate (Pi) transporter 1), *VvPHT2–1* (*V. vinifera* phosphate transporter 2–1), and *VvPHT1–4* (*V. vinifera* phosphate transporter 1–4) [17,18,19,24,25]. Transcript levels of these genes provide insight regarding metabolic activities of NPK, which can be used to foresight nutritional necessity and establish required doses of fertilizer in grape.

Balanced fertilizer is required for economical crops, which can be optimized by scientific methods. Therefore, agriculture production be under by the unscientific use of fertilizer. Residue of excess fertilizer disturbs the balance of soil-nutrients and prevents beneficial growth of plant. Economical crop can be achieved by application of required amount of correct fertilizer type [26,27,28]. Because, crops can respond to NPK fertilizers of the proper rate, time, applied appropriately to soils along with suitable agronomic implementations devoid of other crop limiting restrictions [29].

The aim of this study was to evaluate the response of different commercial NPK fertilizers of different countries based on grape quality, and yield. Furthermore, the present study intended to illuminate gene expression-based practicability and workability of these fertilizers on grape that might be useful for farmers to choose from available NPK sources based on their locality and preferences. Furthermore, the study intended to help commercial industry to formulate new combinations of NPK for potential yield and quality grape.

## 2. Results

### 2.1. Fruit Biochemical Attributes

**Figure 1:**
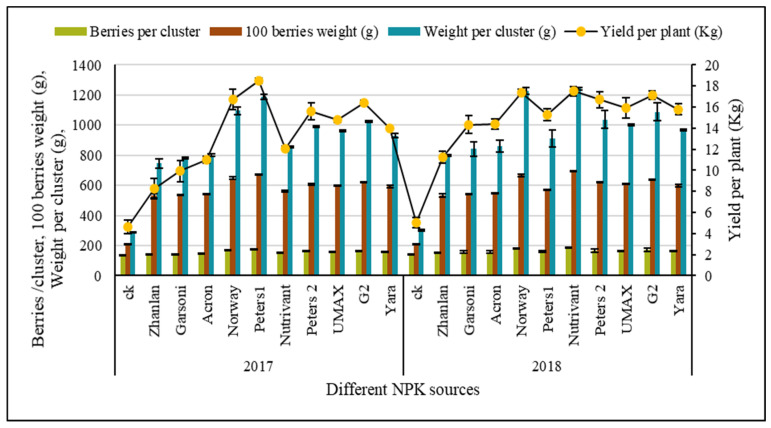
Biochemical attributes [TSS (Brix°), TSS/TA, TA (%)] and yield forming parameters [berries per cluster, 100 berries weight (g), weight per cluster (g) and fruits yield per plant (Kg)] of Kyoho grapes as influenced by the application of various industrial fertilizer combinations (different NPK ratios) during experimental year 2017 and 2018. TSS, TSS/TA, Berries/cluster, 1000 berries weight and weight per cluster are presented on left vertical axis, while TA and yield per plant are presented on right vertical axis of graph. Error bars above mean denote the standard error of three replicates.

At stage L-38, TA%, TSS (Brix°), TSS/TA, and pH were measured from grape berries subjected to different fertilizer combinations. A significant interaction between fertilizer combinations and both years of study was found, so the data are presented separately for both years (2017 and 2018). Figure 1 represents the data regarding TSS, TSS/TA, and TA. Highest TSS was found by the application of Peters 1 in 2017, followed by G2 and Peters 2 in the same year. Application of Norway exhibited the lowest TSS significantly in 2018 as compared to all other treatment combinations as it was 17% less than control (no fertilizer applied) in the same year and 52% less than control in 2017.

Peters 1 and G2 in 2017 and Peters 1 in 2018 depicted the highest TSS/TA, as these fertilizers depicted 29%, 14%, and 28% increases, respectively, as compared to their respective controls. Peters 1 application showed the least TA% during both years of study, while its highest value was recorded by the application of Norway in 2017 (Figure 1).

The application of Peters 1 in 2018 recorded the highest pH in fruits, followed by the same fertilizer in 2017 (Table 1). Norway application in 2017 produced highly acidic fruits, as it showed a minimum pH (17% less than respective control).

### 2.2. Yield Parameters

Years and fertilizer combinations depicted significant interaction regarding the number of berries per cluster, 100 berries weight, weight per cluster, and fruit yield per plant. While the number of clusters per plant was non-significant regarding any treatment applied (Table 2). Control treatment (no fertilizer applied) depicted lowest yield (*p* < 0.5) contributing parameters in both years. Norway application in 2018 and Peters 1 application in 2017 and 2018 recorded significantly highest number of berries per cluster and weight per cluster (Figure 1). Norway and Peters 1 in 2017 and Norway and Nutrivant in 2018 recorded the significantly (*p* < 0.5) highest weight of 100 berries in both years.

Peters 1 recorded the highest fruit yield (18.49 Kg per plant) during the first year of study as a ~4–5-fold increment was recorded compared to control followed by Norway. However, application of Norway resulted in the highest fruit yield during the second year along with Nutrivant, and it showed a 4-fold increment in fruit yield compared to control. Norway was the only fertilizer that resulted in higher and sustained grape yield during both years of study.

### 2.3. Macro- and Micro-Nutrients Contents in Grape Leaves

**Figure 2:**
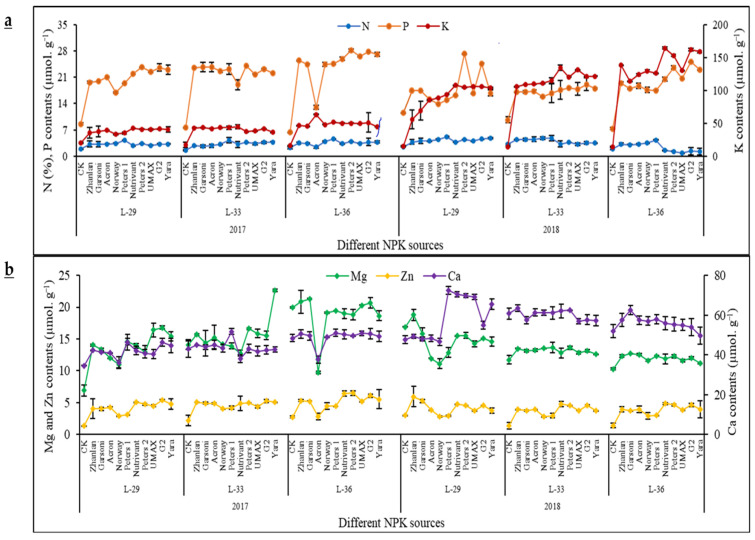
Effect of various fertilizer sources (having different NPK ratios) on (**a**) nitrogen (N; %), phosphorus (P; µmol·g^−1^), potassium (K; µmol·g^−1^), (**b**) calcium (C; µmol·g^−1^), magenssium (Mg; µmol·g^−1^), and zinc (Zn; µmol·g^−1^) measured from grape leaves. N and P contents are presented on left vertical axis while K contents are presented on the right vertical axis of the graph. Error bars above the mean denote the standard error of three replicates.

Control treatment recorded minimum N, P, and K contents in grape leaves at all growth stages under study in 2017 (Figure 2a). Slight fluctuations were recorded regarding N contents by the application of different NPK fertilizers. Maximum was observed in leaves fed with Peters 1. Nutrivant at L-29 and Acron at L-36 were at the same level as the control in 2017. In 2018, Nutrivant, Peters 2, UMAX, G2, and Yara application recorded significantly lower N contents as compared to control at L-33 and L-26. P contents in grape leaves depicted a decline in 2018 as compared to 2017 at L-33 and L-36 stage. The highest P contents (4-fold increase as compared to control) were recorded by the application of Peters 2 in 2017 at L-36 stage. While the aforementioned fertilizer source recorded the highest P contents at L-29 stage in 2018. K contents were more in grape leaves in the second year of study than the first year regardless of the growth stage. During 2017, Acron at L-36 stage recorded maximum K contents among all fertilizer sources. In 2018, K contents were increased in grape leaves with the progression in the growth stage as overall highest K contents were recorded at L-36 stage. Nutrivant application recorded the highest 12-fold increase in K contents as compared to control at L-36.

Calcium contents depicted an increment in 2018 as compared to 2017 regardless of growth stage measured. In 2017, Acron application exhibited a 23% decrease in Ca contents than the respective control at the L-36 stage (Figure 2b). The highest Ca contents (21% and 53% increment than respective control, respectively) were recorded by the application of Peters 1 at L-33 in 2017 and 2018. Overall, less Mg contents were recorded in grape leaves in 2018 as compared to 2017. Acron application exhibited a 2-fold decrease in Mg contents than respective control at L-36 in 2017. Highest Mg contents (57% increment than respective control) were recorded by Yara application regardless of the year of study. Norway application in 2018 recorded a 31% decrease in Mg contents than respective control at L-33. Zn contents remained unchanged during both years of study. The lowest Zn contents were recorded by control (Figure 2b).

### 2.4. Anthocyanin Contents in Grape Peel

Different combinations of NPK fertilizers were applied at three different stages, significantly affecting the anthocyanin contents in grape berry peel. Anthocyanins were determined at L-38 stage. Twenty-five contents were detected in treated grape skin.

#### 2.4.1. Cyanidin Contents

**Figure 3:**
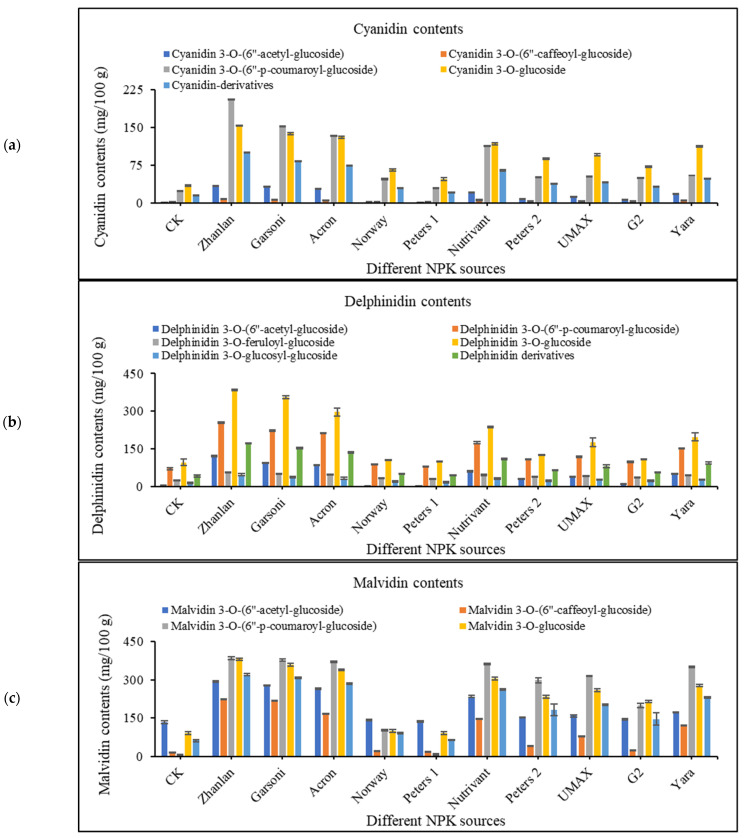
Effect of various fertilizer combinations (different NPK ratios) applied at three growth stages (initiation and termination of fruit setting and mid of veraison stage) on anthocyanin profile- cyanidin (**a**), delphinidin (**b**), and malvidin (**c**) contents in grape berry peel measured at harvest maturity. Error bars above mean denote the standard error of three replicates.

Different cyanidins were measured by HPLC, including; cyanidin 3-*O*-(6″-acetyl-glucoside), cyanidin 3-*O*-(6″-caffeoyl-glucoside), cyanidin 3-*O*-(6″-p-coumaroyl-glucoside), cyanidin 3-*O*-glucoside, and cyanidin derivatives (Figure 3a). Control treatment depicted significantly lower cyanidin contents. Application of different fertilizer sources increased cyanidin contents with significantly highest increase was recorded with the application of Zhanlan as it increased cyanidin contents by ~4–8-folds than control. The lowest increment in cyanidin contents was recorded by the application of Norway and Peters 1 (~2 and 1.5-fold as compared to control, respectively; Figure 3a).

#### 2.4.2. Delphinidin Contents

The response of delphinidin 3-*O*-(6″-acetyl-glucoside), delphinidin 3-*O*-(6″-p-coumaroyl-glucoside), delphinidin 3-*O*-feruloyl-glucoside, delphinidin 3-*O*-glucoside, delphinidin 3-*O*-glucosyl-glucoside, delphinidin derivatives was examined in grape berry peel. Control depicted minimum delphinidin contents while Norway, Peters 1, Peters 2, and G2 were statistically the same with control. The application of Zhanlan and Garsoni showed the highest increment in delphinidin contents with a ~4-fold increase than control (Figure 3b).

#### 2.4.3. Malvidin Contents

Malvidin 3-*O*-(6″-acetyl-glucoside), malvidin 3-*O*-(6″-caffeoyl-glucoside), malvidin 3-*O*-(6″-p-coumaroyl-glucoside), malvidin 3-*O*-glucoside, and malvidin derivatives contents were affected significantly by the application of different fertilizers combinations. Application of Norway and Peters 1 were not able to increase malvidin contents, and both these fertilizers were statistically at the same level as control. However, the approximately 4-fold increase was observed with the application of Zhanlan, Garsoni, and Acron as compared to control (Figure 3c).

#### 2.4.4. Pelargonidin Contents

**Figure 4:**
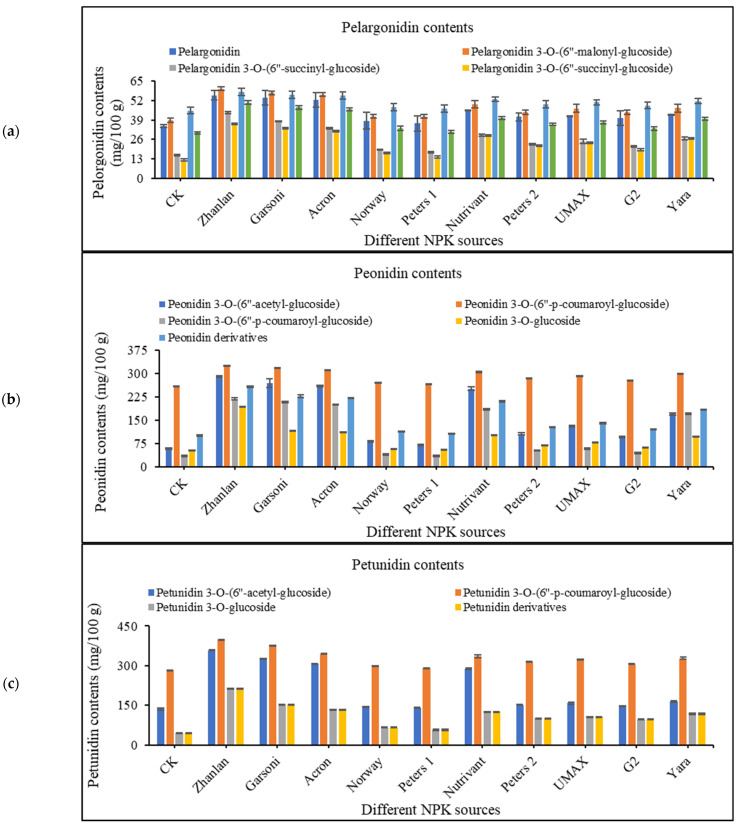
Effect of various fertilizer combinations (different NPK ratios) applied at three growth stages (initiation and termination of fruit setting and mid of veraison stage) on anthocyanin profile- pelargonidin (**a**), peonidin (**b**), and petunidin (**c**) contents in grape berry peel measured at harvest maturity. Error bars above the mean denote the standard error of three replicates.

Pelargonidin contents such as; pelargonidin, pelargonidin 3-*O*-(6″-malonyl-glucoside), pelargonidin 3-*O*-(6″-succinyl-glucoside), pelargonidin 3-*O*-(6″-succinyl-glucoside), pelargonid-in 3-*O*-glucoside, and pelargonidin derivatives were measured to evaluate the differential response of various fertilizers (Figure 4a). A slight increment in pelargonidin contents was observed by the application of fertilizers, with the highest increase was observed with the application of Zhanlan and Garsoni.

#### 2.4.5. Peonidin Contents

Peonidin 3-*O*-(6″-acetyl-glucoside), peonidin 3-*O*-(6″-p-coumaroyl-glucoside), peonidin 3-*O*-(6″-p-coumaroyl-glucoside), peonidin 3-*O*-glucoside, and peonidin derivatives were measured at L-38 stage (Figure 4b). Increment in peonidin contents by the application of Norway, Peters 1, Peters 2, and G2 were the same as the control treatment. While all other fertilizers increased the peonidin contents significantly as compared to control, and all showed the same level of increment.

#### 2.4.6. Petunidin Contents

Different fertilizer combination depicted differential response of petunidin contents such as; petunidin 3-*O*-(6″-acetyl-glucoside), petunidin 3-*O*-(6″-p-coumaroyl-glucoside), petunidin 3-*O*-glucoside, and petunidin derivatives (Figure 4c). Application of Norway and Peters 1 could not increase the petunidin contents as compared to other fertilizers, and these were at the same level of control. While the application of Zhanlan depicted highest (41% as compared to control) increment in petunidin contents.

### 2.5. Relative Expression of Different Genes Understudy

Relative transcript levels of genes related to nitrogen, phosphorus, potassium, and anthocyanin were observed during 2018 at three different growth stages (L-29, L-33, and L-36) and three different parts (leaves, peel, and pulp).

#### 2.5.1. Relative Expression of Nitrogen Pathway Genes

**Figure 5:**
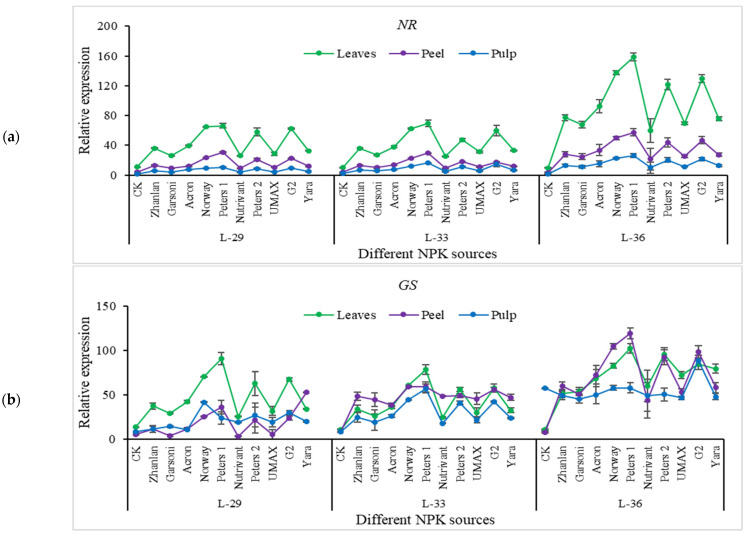

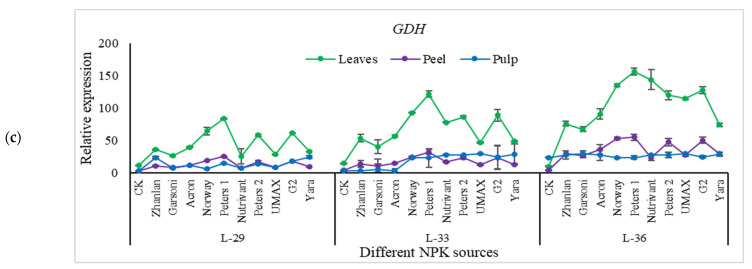
Relative expression of *NR* (**a**), *GS* (**b**), and *GDH* (**c**) in grape leaves, fruit peel and pulp as influenced by various fertilizer sources (having different NPK ratios) applied at three different growth stages (initiation and termination of fruit setting and mid of veraison stage). Error bars above mean denote the standard error of three biological and technical replicates.

**Figure 6:**
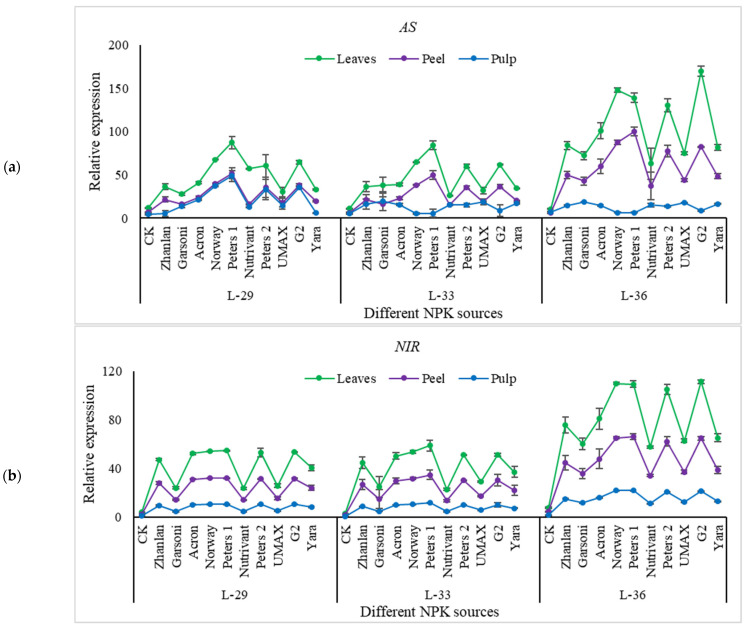
Relative expression of *AS* (**a**), and *NIR* (**b**) in grape leaves, fruit peel and pulp as influenced by various fertilizer sources (having different NPK ratios) applied at three different growth stages (initiation and termination of fruit setting and mid of veraison stage). Error bars above mean denote the standard error of three biological and technical replicates.

Nitrogen pathway genes, including; NR, GS, GDH, AS, and NIR were studied for transcriptional analysis from grape leaves, peel, and pulp. NR relative expression depicted an increasing trend with progression in growth; however, this increase was more evident in leaves as compared to peel and pulp. An 11-18-fold increase in NR transcript level in grape leaves was observed by the application of Peters 1 at L-33 and L-36 as compared to control (Figure 5a). Nutrivant depicted minimum relative expression among all fertilizer sources in grape leaves, and fruit peel and pulp. In peel, this gene exhibited a 25-fold increase in relative expression as a result of Peters 1 application at L-36. Relative expression remained unchanged in fruit pulp for other fertilizers except for Peters 1, which showed an increase in transcript level. Peters 1 application exhibited an increase in GS transcript level in leaves and peel. GS showed higher transcript levels at stage L-36 in leaves and peel (Figure 5b). While the application of G2 exhibited highest transcript levels of GS at L-36 stage. GDH transcript levels showed an increasing trend with the progression in the growth stage. Application of Norway showed 56-fold increase in GDH transcript levels than control at L-36 in leaves. Relative expression of GDH did not show any increase in relative expression in fruit pulp. While in fruit peel, Norway and Peters 1 depicted an increase in transcript levels of GDH than other fertilizers (Figure 5c). Relative expression of as was higher in leaves and peel at stage L-36 than in other stages. Peters 1 depicted maximum relative expression of AS at stage L-29 and L-33 in leaves and peel. At stage L-36, the application of G2 resulted in the highest transcript levels of as compared to other fertilizers (Figure 6a). Transcript levels of NIR showed the same trend as AS. While maximum transcript levels were recorded at L-36 by the application of Norway, Peters 1, Peters 2, and G2 in leaves, peel, and pulp (Figure 6b).

#### 2.5.2. Relative Expression of Phosphorus Pathway Genes

**Figure 7:**
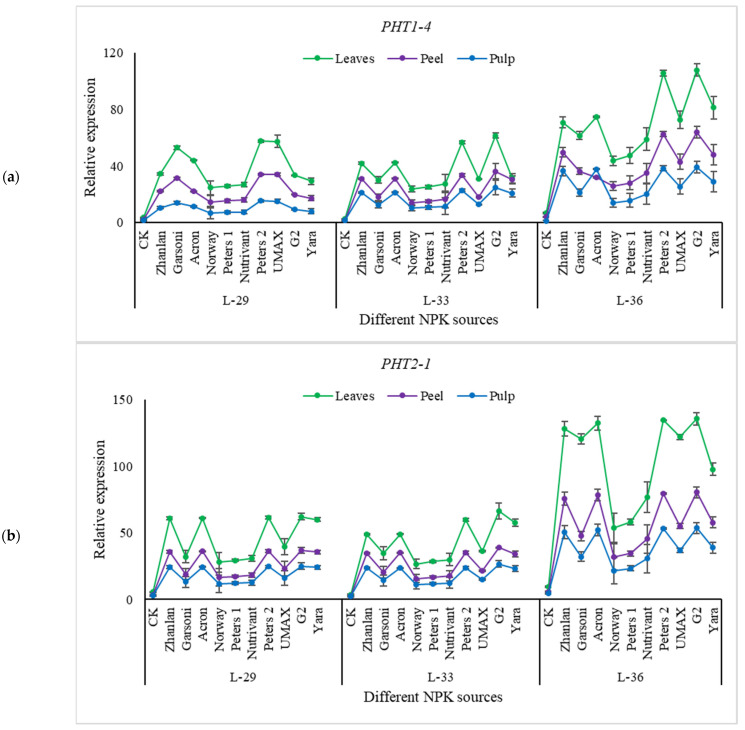
Relative expression of *PHT1-4* (**a**), and *PHT2-1* (**b**) in grape leaves, fruit peel and pulp as influenced by various fertilizer sources (having different NPK ratios) applied at three different growth stages (initiation and termination of fruit setting and mid of veraison stage). Error bars above mean denote the standard error of three biological and technical replicates.

**Figure 8:**
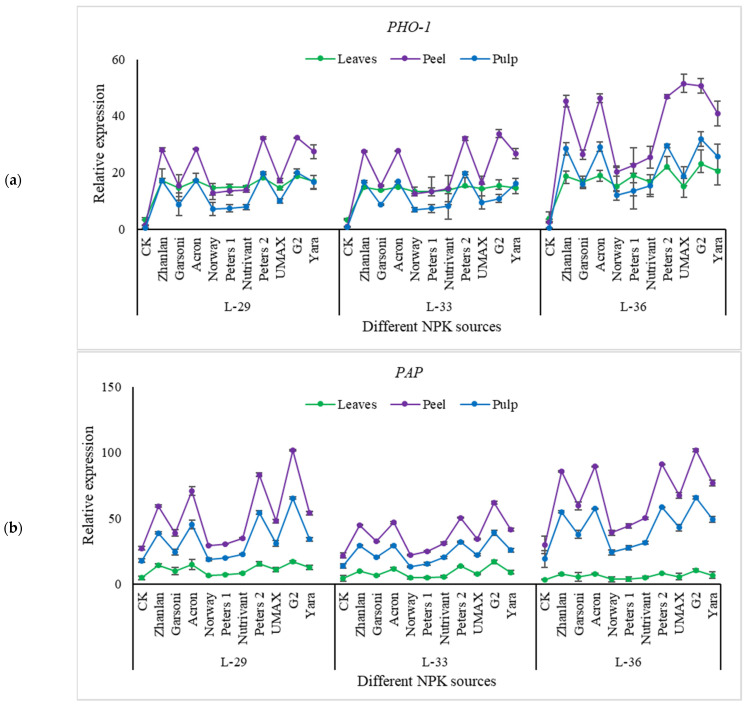
Relative expression of PHO-1 (**a**), and PAP (**b**) in grape leaves, fruit peel and pulp as influenced by various fertilizer sources (having different NPK ratios) applied at three different growth stages (initiation and termination of fruit setting and mid of veraison stage). Error bars above mean denote the standard error of three biological and technical replicates.

Transcript levels of PHT1-4, PHT2-1, PHO-1, and PAP were studied in grape leaves, fruit peel, and pulp at L-29, L-33, and L-36. Relative expressions of PHT1-4 and PHT2-1 depicted an increasing trend with the progression of growth stages as highest transcript levels were measured at L-36 (Figure 7). Peters 2 depicted maximum transcript levels PHT1-4 and PHT2-1 in leaves, peel, and pulp regardless of the growth stage, except PHT2-1 at stage L-36. While G2 was at same transcript level of PHT1-4 at stage L-36, relative expressions of PHO-1 were higher in grape peel and as compared to leaves and pulp (Figure 8a). Peters 2 and G2 at L-29 and L-33 and Peters 2, G2, UMAX, Yara, Acron, and Zhanlan at L-36 depicted higher transcript levels of PHO-1 in the peel. Transcript levels of PAP were higher in fruit peel and pulp and compared to leaves. While L-33 showed relatively less relative expression of PAP as compared to other stages (Figure 8b). Petters and G2 showed maximum relative expressions of PAP in peel and pulp regardless of the growth stage.

#### 2.5.3. Relative Expression of Potassium Pathway Genes

Relative expressions of HKT1, SIRK, SORK, KUP1, and KUP2 were observed in grape leaves, fruit peel, and pulp at three different stages (L-29, L-33, and L-36).

**Figure 9:**
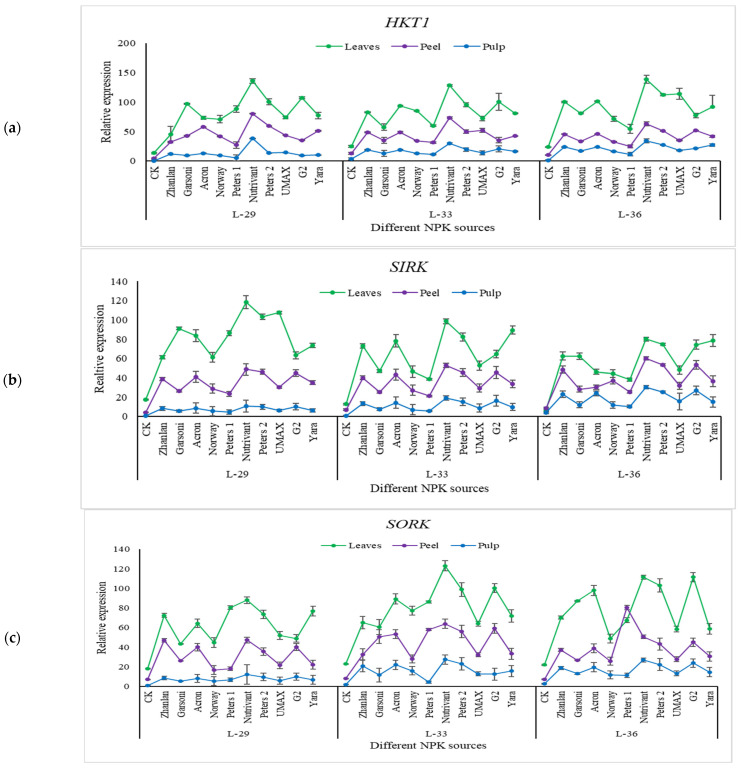
Relative expression of *HKT1* (**a**), *SIRK* (**b**), and *SORK* (**c**) in grape leaves, fruit peel and pulp as influenced by various fertilizer sources (having different NPK ratios) applied at three different growth stages (initiation and termination of fruit setting and mid of veraison stage). Error bars above mean denote the standard error of three biological and technical replicates.

**Figure 10:**
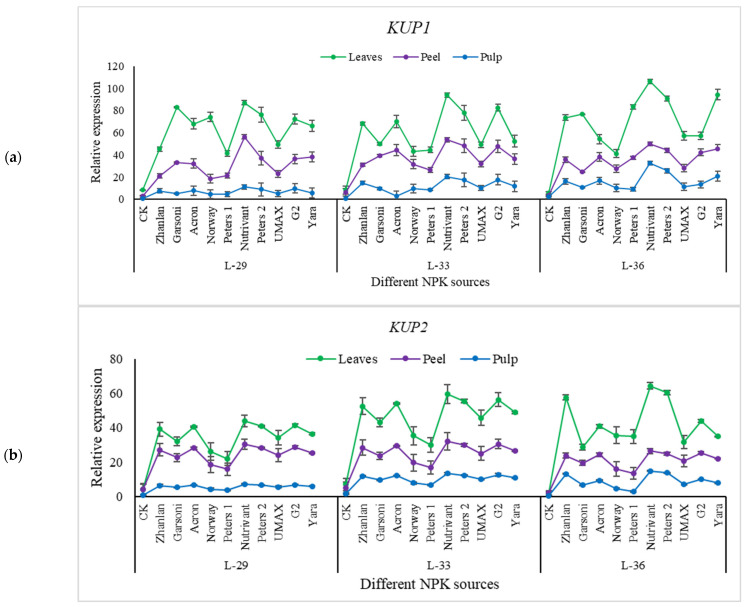
Relative expression of *KUP1* (**a**), and *KUP2* (**b**) in grape leaves, fruit peel and pulp as influenced by various fertilizer sources (having different NPK ratios) applied at three different growth stages (initiation and termination of fruit setting and mid of veraison stage). Error bars above mean denote the standard error of three biological and technical replicates.

Relative expressions of HKT1 remain the same at all growth stages, except for pulp. Nutrivant application resulted in maximum HKT1 relative expression among all fertilizers with a ~6-fold increase as compared to respective controls (Figure 9a). SIRK transcript levels were maximum at L-29 as compared to other growth stages, and leaves showed maximum transcript level compared to peel and pulp (Figure 9b). SORK relative expressions were lower at L-29 as compared to other stages. Maximum transcript levels of SORK were recorded in leaves followed by peel and pulp (Figure 9c). Maximum relative expressions of SIRK and SORK were observed by the application of Nutrivant regardless of the growth stage. Relative expressions of KUP1 remained unchanged at all stages (Figure 10a). However, transcript levels of KUP2 were higher at L-36 than in other stages (Figure 10b). Nutrivant increased the relative expressions of KUP1 and KUP2 more as compared to other fertilizers in leaves, peel, and pulp.

#### 2.5.4. Relative Expressions of Anthocyanin Structural Genes

PAL, CHS, F3′H, F3′5′H, DFR, LDOX, UFGT, OMT, and GST were studied for differential relative expressions in grape leaves, fruit peel, and pulp at L-29, L-33, and L-36.

**Figure 11:**
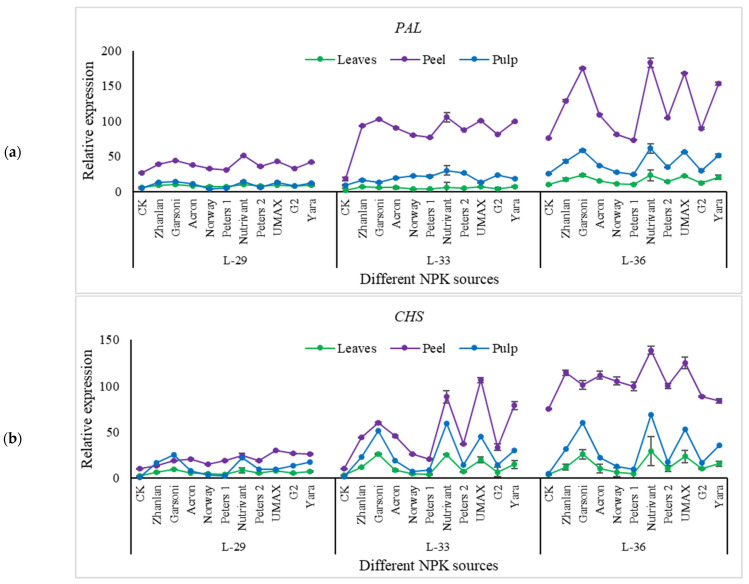

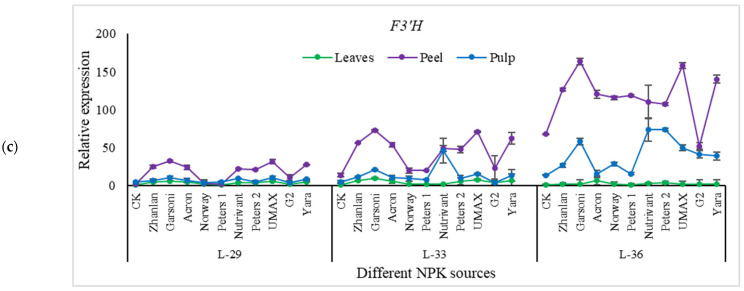
Relative expression of *PAL* (**a**), *CHS* (**b**), and *F3′H* (**c**) in grape leaves, fruit peel and pulp as influenced by various fertilizer sources (having different NPK ratios) applied at three different growth stages (initiation and termination of fruit setting and mid of veraison stage). Error bars above mean denote the standard error of three biological and technical replicates.

**Figure 12:**
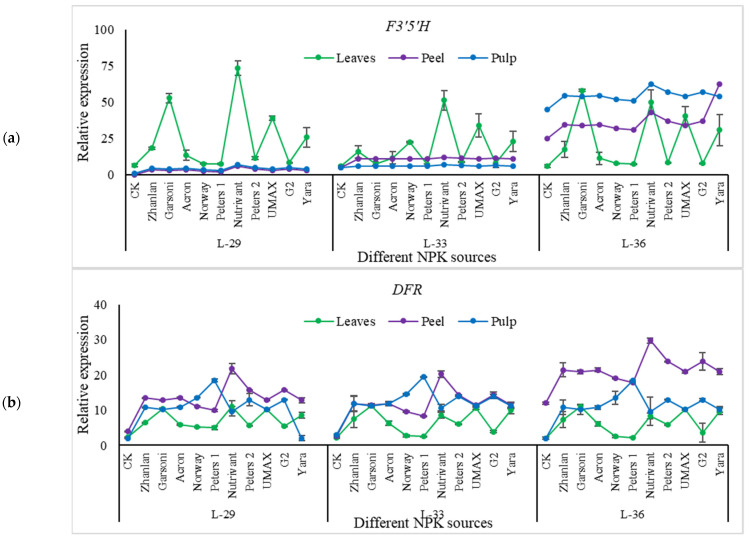

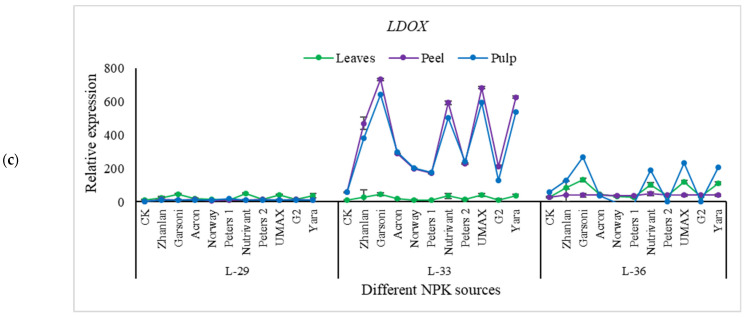
Relative expression of *F3′5′H* (**a**), *DFR* (**b**), and *LDOX* (**c**) in grape leaves, fruit peel and pulp as influenced by various fertilizer sources (having different NPK ratios) applied at three different growth stages (initiation and termination of fruit setting and mid of veraison stage). Error bars above mean denotes the standard error of three biological and technical replicates.

**Figure 13:**
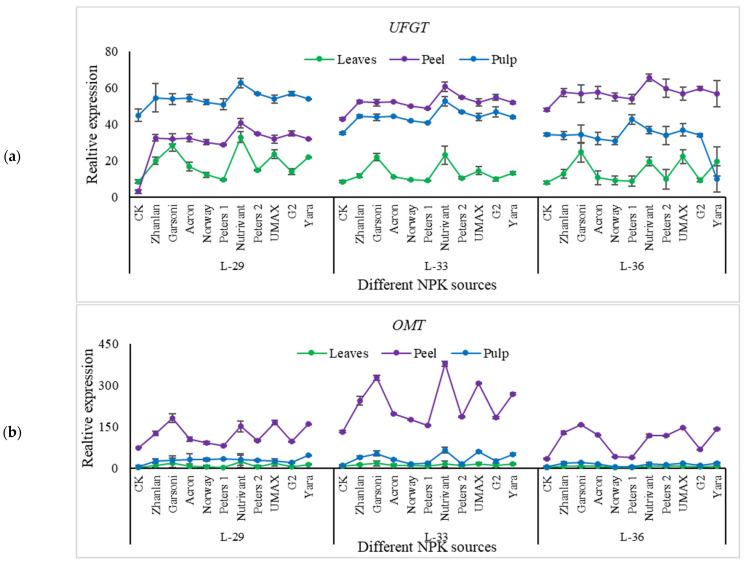

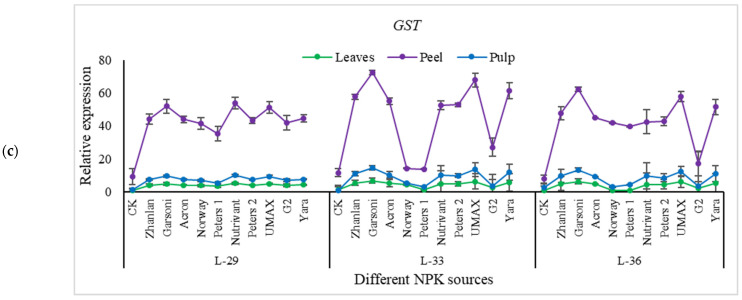
Relative expression of *UFGT* (**a**), *OMT* (**b**), and *GST* (**c**) in grape leaves, fruit peel and pulp as influenced by various fertilizer sources (having different NPK ratios) applied at three different growth stages (initiation and termination of fruit setting and mid of veraison stage). Error bars above mean denote the standard error of three biological and technical replicates.

Relative expressions of PAL, CHS, F3′H, DFR, OMT, and GST were higher in fruit peel as compared to leaves (Figure 11, Figure 12 and Figure 13). Peters 1 and Norway resulted in the lowest PAL transcript levels at L-36, and it was the same as control. While Nutrivant and Garsoni recorded the highest relative expressions at the same stage. L-36 recorded the highest PAL and CHS relative expressions as compared to other stages (Figure 11a,b). Nutrivant caused a maximum increase in CHS relative expressions at L-36 among all fertilizers. While the application of Garsoni and UMAX resulted in the highest F3′H transcript levels than other fertilizers at the same stage (Figure 11c). Transcript levels of F3′5′H were highest in leaves at L-29 and L-33 than peel and pulp (Figure 12a). While the application of Nutrivant resulted in highest relative expressions of F3′5′H at stage L-36 in the pulp. DFR relative expression in peel was higher at L-36 than leaves and pulp, while the other two stages did not exhibit any difference in DFR relative expressions in leaves, peel, and pulp (Figure 12b). Nutrivant application resulted in the highest DFR relative expression than other fertilizers at L-36. Relative expressions of LDOX were maximum in peel at L-33 as compared to leaves and pulp. Garsoni application resulted in a maximum increase in LDOX transcript levels than other fertilizers with 12-fold increase as compared to respective control (Figure 11c). At stage L-33 and L-36, relative expressions of UFGT were higher in the peel as compared to leaves and pulp while these were higher in the pulp at L-29 (Figure 13a). Nutrivant application depicted a maximum increase in UFGT transcript levels as compared to other fertilizers at all stages in fruit peel and pulp. Relative expressions of OMT were significantly higher in the peel as compared to leaves, and pulp and maximum were recorded at L-33. Application of Garsoni at L-29 and Nutrivant at L-33 resulted in the highest transcript levels as compared to other fertilizers with 2 and 3-fold increase as compared to respective controls, respectively (Figure 13b). GST transcript levels remained unchanged in leaves and pulp at all stages (Figure 13c). Relative expressions of GST were significantly higher in the peel as compared to leaves and pulp at all stages. Nutrivant at L-29, Garsoni at L-33 and L-36 depicted highest transcript levels than other fertilizers with 6, 7, and 21-fold increases as compared to respective controls, respectively.

## 3. Discussion

In this study, an investigation of grapewine’s N, P, and K uptake and metabolism genes response to applied fertilizers of different NPK ratios was carried out. There are different types of N, P, and K fertilizers and these types have exclusive characteristics that is specific for different crops. Besides, yield and quality parameters of grapes can be optimized by the application of specific fertilizer combinations [30,31,32].

Nevertheless, it is challenging to forecast the precise nutritional requirements of grapes on various developmental stages. Internal and external factors influence these diagnostic techniques, and these are less sensitive to be employed as fertilization strategies. Though by exploring the transcript levels of NPK uptake and metabolism genes, the precise nutrient requirements of various developmental stages could be find out, and adequate fertilizer absorption time can be predicted, consequently permitting us to fertilize with the accurate nutrient type at the right time. At this point, the advanced effects can be included since berry ripening indicators such as; °Brix index, grape juice pH value, sugar to acidity ratio, along with the berry weight and fruit yield at harvest, were expressively affected by different fertilizers application. Consequently, the experiential effects of N on anthocyanin contents can be related to alteration in anthocyanin biosynthetic pathway regulation.

### 3.1. Various NPK Sources

NPK fertilizers having different NPK ratios from various companies were used in this study to evaluate their effects on anthocyanin contents, biochemical parameters, mineral content, related gene transcript levels, and yield contributing parameters of grapes. Among the ten fertilizers used, Acron, Norway, and Peters 1 had differential ratios of NPK. Acron and Norway had more nitrogen as compared to phosphorus and potassium, while Peters 1 had more potassium concentration. Plant nutrition diagnosis, soil testing, and formulation of fertilizers are the main focus of crop fertilization research [33,34,35,36,37]. These techniques permit us to monitor the soil nutritional status, nutrient uptake, and metabolism stages in plants throughout growth and development, consequently providing an absolute guiding principle for fertilizer application.

### 3.2. Chlorophyll and Carotenoids Contents

Chlorophyll and carotenoids were increased by fertilizers of the higher ratio of NPK. Nutrivant and Acron application recorded the highest and at par chlorophyll contents in 2018 at L-29 and L-33 stage. Chlorophylls (principal light-absorbing pigments) are the vital components of the plant’s photosynthesis. A drastic decrease in total chlorophyll content is obvious in plants with less N compared to other treatments. A probable clarification for the deterioration in chlorophyll during N famishment is that chlorophyll molecules structure has four N atoms. Thus, it becomes complicated for the cell’s organelles to manufacture these compounds in nitrogen source absenteeism [38]. Mineral nutrition controls the chlorophyll and carotenoid synthesis [39]. Plant stability in unfavorable conditions is facilitated by green pigments in leaves that is dependent upon P concentration [40]. Nevertheless, the simplification of biochemical characteristics [41] and pigment molecules biosynthesis depend on the optimal P uptake [42]. Better plant growth and an increase in total chlorophyll contents was observed in apricot seedlings under optimal P conditions [43]. An increase in biomass production, as well as carotenoid content was observed in blue-green alga (Spirulina platensis) by P application [44]; however, higher P concentrations were responsible for the reduction of chlorophyll content in blue-green alga (*Azolla pinnata*) [45]. Another study reported a decline in chlorophyll content in maize under P deficiency [46].

### 3.3. Leaf Macro- and Micro-Nutrients Levels

Leaf nitrogen was highest in plants applied with Petrer1 due to the highest amount of nitrogen in this fertilizer. In both years of study, the maximum leaf N concentrations were detected in grapevines applied with the higher N application. This most possibly occurred as fertigation endorsed the urea dissolution by accelerating urease hydrolysis. This lead to ammonium carbonate formation, which decomposes quickly, forming hydroxyl, carbonate, and ammonium. Later on, through biological oxidation, this might be converted into nitrite (NO_2_^−^) and nitrate (NO_3_^−^) [1]. Moreover, irrigation water can help the migration of nitrate in root zone subsequent to urea application [47], and mass flow might also help in movement of N from the soil solution to the root zone [48]. Increasing N fertilizers tended to increase N content in leaves of grapes. The results are in accordance with those obtained in earlier studies [40,41].

Furthermore, in orange leaves an increase of N application also caused a decline in N concentration [49]. The highest P contents (4-fold increase as compared to control) were recorded by the application of Peters 2. Nutrivant application recorded the highest 12-fold increase in K contents as compared to control at L-36. The N-fertilizer application alone did not affect the concentration of Ca, Mg, and K in apple leaves and fruits, but increased these elements when combined with P and K in tomato [50]. In this study, balanced N, P, and K increased above mentioned nutrients is similar to previous studies in which up to a certain quantity, fertilizer increased nutrients in leaves after that no increase was observed [51].

### 3.4. Berry Yield

By the results of the study, it is clear that the berry yield by Norway and Nutrivant application is at the same level, and the same fertilizer recorded the highest fruit yield. Higher berry weight ultimately increased yield by highest N application along with P and K. Berry weight and yield tended to increase with increasing N ratio in combined fertilizer according to the previous findings [13,52,53]. In this study number of clusters and berries is resulting in a higher yield, which coincides with previous findings [54]. The solitary effects of macro-nutrient fertilizers showed that the yield and yield components were proved to more K sensitive as compared to N and P fertilizers, and the results specified that yield was increased by increasing the application of N and K [55,56]. Furthermore, it was found that only K fertilizers significantly affected the number of seeds per pod [56]. Previously, research revealed that K application is a practicable approach for yield improvement [57]. Specifically, the N, P, and K combinations showed additional yield effects compared to the sole interaction effects of N, P, and K fertilizers [58,59].

### 3.5. Fruit Biochemical Attributes

The highest values of acidity and lowest TSS were found in grapes applied with Zhanlan having comparatively high NPK nitrogen. The possible explanation of these results is that the grapevines absorbed greater amounts of mineral N from the soil, that usually rouse shoot growth, causing the lower frequency of solar rays reaching into the grapevines. The more shading of the grape clusters inside the grapevine hampers canopy aeration from modifying temperature, that can lower sugar contents and upsurge the berry acidity [51]. The highest TSS was observed in grapes applied with a fertilizer containing the highest potassium ratio. This is hypothesized that increasing K fertilization enhanced sugar transportation into the berries [60].

### 3.6. Anthocyanin Contents

Anthocyanin belongs to flavonoid family of polyphenol phytochemicals. It gives bright red-orange to blue-violet color to numerous fruits, as well as vegetables also. Anthocyanin exists in the form of aglycone bind to different sugar moiety in the plants. Vital anthocyanin (stored in vacuoles) comprises of cyanidin, delphinidin, peonidin, pelargonidin, petunidin, and malvidin [61,62]. In this study, among fertilizers of different N, P, and K ratio, fertilizer of low N increased anthocyanin. Norway having more nitrogen recorded less anthocyanin content in fruit peel, while Zhanlan and Gorsani recorded relatively more anthocyanin contents in this study. Similarly, the anthocyanin content of grape skin was discovered to intensify at a small N supply compared with high N application [63]. Inorganic compounds (ammonium and potassium nitrate) perform a momentous part in plant metabolism for the anthocyanin production and additional secondary metabolites [64]. Another study [65] stated that altering the proportion of ammonium to potassium nitrate caused in a expressively advanced buildup of anthocyanin in apple. Anthocyanin biosynthesis is frequently affected by numerous environmental factors, such as light, temperature, irrigation, as well as soil N content [63,66,67,68]. High nitrogen vine status may affect anthocyanin in many ways, such as it can straight affect metabolism of anthocyanin, deferring berry development and ripening and enhancing the pulp to skin ratio, in that way reducing berry anthocyanin by a meek dilution effect [69].

### 3.7. Anthocyanin and NPK Pathway Genes

The structural genes of anthocyanin and potassium-pathway in the present study showed higher expression levels by the application of Nutrivant that has NPK in equal ratio. The Nutrivant depicted higher K contents in leaves, and yield contributing parameters were higher by the same fertilizer application. The same fertilizer showed increased anthocyanin contents as compared. The structural genes indoctrination of the enzymes accountable for each catalytic step have been categorized, and the knowledge of corresponding molecular regulation has significantly proceeded over past two decades [70,71,72,73]. In Arabidopsis and tomato, the N supply effects on the regulation pathway were explored previously [74,75]. In the present study, of grape berries, it is clear that the development of the anthocyanin contents is firmly associated with the biosynthetic genes expression pattern. There are two classes of enzymes elaborating the biosynthesis pathways-early genes that comprise of- phenylalanine ammonia lyase (PAL), cinnamate 4-hydroxylase (C4H), 4-coumaroyl:CoA-ligase (4CL), chalcone synthase (CHS), chalcone isomerase (CHI), flavanone 3-hydroxylase (F3H), flavonoid 3′5′-hydroxylase (F3′H), and late genes-dihydroflavonol reductase (DFR), anthocyanidin synthase (ANS), also called leucoanthocyanidin dioxygenase (LDOX) and UDP glucose:flavonoid-3-*O*-glucosyltransferase (UFGT) [76]. A previous study has described the ammonium nitrate and potassium nitrate effects on pathway genes of anthocyanin [77]. In *Solanum lycopersicum* leaves, nitrogen depletion caused an increase in transcript levels of PAL, CHS, and F3H [78]. Correspondingly, in Arabidopsis, the enhanced transcript levels of PAL, CHS, F3H, DFR, and DOX genes were witnessed by the low N concentration [79].

Different ratios of NPK play various roles in improving gape attributes. NPK fertilizer should be chosen on the basis of required parameters in grape. Although applying fertilizers based on gene expression data has been confirmed by the findings of our study and it can be regarded as a viable and applied fertilization approach, further appraisal of various fertilizer forms, mixtures, and doses rendering to transcript levels knowledge will permit us to accomplish a wider goal. Furthermore, that strategy will allow us to forecast the nutritional needs of plants. Additionally, fertilizers application with the optimal type at the most reasonable application rate will also be convenient. The present research can contribute to improving traditional fertilization practices, and it can serve as one of the important findings concerning the fertilizer application in vineyards. Despite the growth conditions were the same for all plants under study; however, the molecular makeup of plants can be affected by many other cultural practices and environmental factors.

## 4. Materials and Methods

### 4.1. Experimental Design

**Figure 14:**
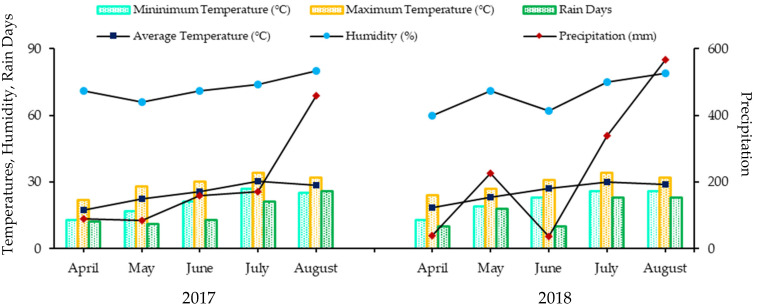
Mean monthly weather data of experimental site (Lishui, Nanjing, China) during April–August in 2017 and 2018. Left vertical axis depicting minimum, maximum, and average temperature (℃), humidity (%), rain days, while precipitation (mm) is presented on right vertical axis. Data collected from Nanjing Institute of Meteorology, Jiangsu, China.

This study was designed to evaluate the impact of commercial NPK fertilizers on grape physical, physiological, fruit quality, biochemical, anthocyanins profiles, and gene expressions on Kyoho grape (*V. vinifera* × *V. labrusca*) in 2017 and 2018. Experimental site was Grape Experimental Vine-Yard, Jiangsu Academy of Agricultural Sciences (31°62′ N/119°18′ E), Baima, Lishui District, Nanjing, China. Weather data of the experimental site was collected from Nanjing Institute of Meteorolgy, Nanjing, Jiangsu, China (Figure 14).

Ten commercial NPK fertilizers of different companies each 20 gm were spread on soil around plant surface under canopy on three stages, L-29, L-33, and L-36, in accordance to the E-L system [80] followed by irrigation. According to the E-L system, fruit setting period ranges from L-27–L-34, and the veraison stage ranges from L-35–L-37. Therefore, the initiation (L-29) and termination (L-36) of fruit setting stages were selected for fertilizer application along with the mid of the veraison stage (L-36). One control (CK) treatment was maintained for comparison in which no fertilizer was applied. Pilot trial was performed to evaluate the response of various doses (10, 20, 30, 40, and 50 gm per plant) of these fertilizers on 15 grape vines. Yield and yield-related parameters were recorded. The dose of 20 gm fertilizer per plant was selected on the basis of yield and economics. Fruits were harvested at L-38. Leaf and fruit samples were collected after 72 h of application and stored at −80 °C after dipping in nitrogen for subsequent analysis. Fruits for anthocyanins and genes studies were peeled to store both skin and flesh individually before storing at −80 °C. The detail of the experimental design is given in Table 3.

### 4.2. Yield Components

Ripe grape berries were harvested for fruit quality and quantity indices at L-38 (harvest stage). Yield components include number of berries per cluster, total fruit weight per cluster (g), 100 berries weight (g) yield per plant (Kg), and number of clusters per plant. Weighed at least 10 clusters of grape samples for each product to calculate the average weight of the cluster, and used 100 berries per cluster to calculate the average weight of the berries.

### 4.3. Nutrients Determination in Leaves (ICP-OES)

Grape leaf samples were collected to assess the amounts of plant nutrients at (E-L 29), (E-L 33), and (E-L 36) stages. Leaf samples were collected after 96 h of NPK application, for nutrient examination. Leaves were collected to the opposite side of a ring. The blade of the leaf was immediately removed from the petiole, dried under the shade then stored for 48 h in an oven at 65 °C. The samples dried were ground into a fine powder for analysis using a hand mill. Powdered leaves weighted 0.1gm were shifted into digestion tubes of ETHOS Touch control advanced microwave lab stations of Milestone microwave laboratories (Gerhardt, Brackley, UK). After that 5mL of concentrated nitric acid (HNO_3_) was added into tubes, and then placed for digestion for 2 h in above mentioned machine. After cooling samples at room temperature, the final volume of each sample was adjusted 10 mL with distilled water. Specific ion content was measured using an inductively coupled plasma optical emission spectrometer (ICP-OES, Perkin Elmer Optima 2100 DV, Waltham, MA, USA) (Life Sciences College, Nanjing Agriculture University, China). The blank samples were prepared and processed by adding 5 mL of concentrated nitric acid to an empty digestive vessel while standard solutions of each element were prepared in different concentrations [30].

### 4.4. Nitrogen Determination in Leaves

The leaves were washed with distilled water and dried in an oven with forced air circulation at 65 °C until reaching constant weight. The leaves were then ground in a Wiley-type mill and passed through a 2 mm mesh sieve. Sulfuric acid digestion of the leaves was carried out according to the methodology proposed earlier [81]. Total N was determined by Micro-Kjeldhal digestion followed by ammonia distillation (Tecnal, São Paulo, TE-0363, Brazil) [82].

### 4.5. Biochemical Parameters

Fresh 100 g fruit was squeezed to determine juice total soluble solids (TSS), measured using a portable hand-held dialyzer digital Atago PAL-1 meter (Atago Co. Ltd., Saitama, Japan), and the titratable acidity (%) was titrated with 0.1 mol/L NaOH. pH was measured with Mettler Toledo FE20 Desktop pH Meter (Mettler Toledo Instruments Co. Ltd., Shanghai, China) [83].

### 4.6. Extraction of Fruit Skin’s Individual Anthocyanins by UPLC

Grapes fruit skin samples were analyzed at the harvest stage (L-38) to determine anthocyanin components.

Around 50 berries were picked from each pack, skins were peeled and powered with liquid nitrogen, then frozen for 24 h at −80 °C in FreeZone 4.5 Plus LABCONCO until weight was constant [84].

Peel 0.50 g of powder was blended with 10 mL of solution HCl/methanol/water (1:80:19, *v*/*v*/*v*, 1 mol/L). The mixture was collected for 10 min under 100 Hz ultrasonic waves, and then shaken for 30 min at 25 °C in the dark at a rate of 150 rpm. Then, using a high-speed, refrigerated centrifuge, the sample was centrifuged at 10,000 rpm and 4 °C for 10 min, and the supernatant was collected. Then, we re-extracted the residue four times. Finally, both supernatants were dried with a rotary evaporator, then re-dissolved with mobile phase A in 10 mL. Prior to UPLC analysis, all extracts were filtered with a filter of 0.45 μm.

A 2μL solution aliquot with a flow rate of 0.4 mL/min was injected into the UPLC column (2.1/100 mm ACQUITY UPLC BEH C18 column containing 1.7 μm particles). Buffer A composed of 0.1 per cent formic acid in water while buffer B was 0.1 per cent formic acid in acetonitrile. The buffer B gradient was 5 percent of buffer B for 2 min, 5−95 per cent for 15 min, and 95 per cent for 2 min. Mass spectrometry was performed with MSe acquisition mode using an electrospray source, with a selected mass range of 50−1200 *m*/*z* in both positive and negative ion mode. Leucine-enkephalin (*m*/*z* 556.2771 for positive ion mode, 554.2615 for negative ion mode) was allowed for recalibration to the lock mass options. The ionization parameters were: the capillary voltage was 2.5 kV, the colliding energy was 40 eV, the source temperature was 120 °C and the temperature of the desolvation gas was 400 °C. Using MASslynx 4.1, the data acquisition and processing was carried out. Malvidin-3-*O*-glucoside (Extrasynthese, Genay, France) was used as a typical external standard, and it quantified other anthocyanin components.

### 4.7. Total RNA Isolation, cDNA Synthesis, and Gene Expression

In this study, 24 genes (Table 4), including anthocyanins pathway structural genes, potassium, nitrogen, and phosphorous pathway genes were included.

#### 4.7.1. RNA Isolation and cDNA Synthesis

According to the research [83] total RNAs were isolated using a CTAB procedure. The first-strand cDNA was then synthesized from 1 μg total RNA with P1[an oligo(dT)20 primer], P02(a random primordial), and Invitrogen (Carlsbad, CA, USA) SuperscriptIII RNAse H-RT package, AS instructed by the manufacturer. For RT-PCR the cDNA has been diluted at 1:10. RT-PCR was performed using CFX96 Real-Time PCR Detection System (Bio-Rad, Hercules, CA, USA).

#### 4.7.2. qPCR and Gene Expression Analysis

The volume of the reaction mixtures was 10 μL, including 5 μL of SYBR Green Supermix (Bio-Rad), 2 μL of diluted cDNA, and 0.2 μL of each primordial. Each pair of qRT-PCR primers had been validated with this pair of primers by cloning and sequencing the RT-PCR product. Each priming pair’s efficiency was quantified using serial dilution of a PCR product. All biological samples were tested in triplicates. Ubiquitin and EF1ÿ have been used as internal standards to normalize the expression. Expression levels were calculated on the basis of the 2^−ΔΔCt^ method and each control sample was selected as a reference.

### 4.8. Statistical Analysis

Experiment was laid out using randomized complete block design with three replicates. All the fertilizers under study were randomized in three blocks on above mentioned growth stages. SPSS version 15.0 (SPSS, Chicage, IL, USA) was used to construct ANOVA and statistical evaluation of result at *p* < 0.05 level for yield related attributes biochemical parameters, nutrient levels in leaves, and anthocyanin contents in berries. Gene transcript level study was comprised of three biological and technical replicates. Up and down regulation of transcript levels were determined by *t*-test (*p* < 0.5) and fold change. Graphs were drawn using Microsoft Excel 2019.

## 5. Conclusions

In this research, we applied fertilizers of different N, P, and K ratios on three stages to evaluate impact of these fertilizers on grape in field for two years. We found different ratios have different impact on leaf, fruit parameters, and molecular mechanisms, i.e., high nitrogen increased yield might be because nitrogen metabolism genes were upregulated. If the grape yield is the ultimate goal to be achieved, then Norway must be considered because it performed better and sustained the grape yield on both years. However, decreased anthocyanin contents by the application of the highest nitrogen-containing Norway fertilizer is not a desirable character for grape quality. Though Zhanlan application increased anthocyanin profile of grapes, fruit yield cannot be compromised just for anthocyanin increase. Nevertheless, Nutrivant application not only increased the grape yield, but depicted a moderate increase in the anthocyanin profile as well. That depicts its ability to enhance yield and grape quality simultaneously. This article suggested that viticulturists select Nutrivant as potential fertilizer sources. Fertilizer industries may consider the result of this study to design more specified fertilizers for grape vines.

## Figures and Tables

**Table 1:** Effect of various fertilizer combinations (different NPK ratios) on pH of grapes berries recorded during experimental years 2017 and 2018.

Fertilizer Source	pH of Grape Berries	
	2017	2018
CK	5.6 ± 0.036 ^e^	5.98 ± 0.008 ^c^
Zhanlan	5.39 ± 0.026 ^fg^	5.84 ± 0.028 ^d^
Garsoni	5.14 ± 0.052 ^i^	5.74 ± 0.050 ^d^
Acron	4.93 ± 0.017 ^j^	5.48 ± 0.061 ^f^
Norway	4.69 ± 0.005 ^lm^	5.08 ± 0.026 ^i^
Peters1	4.62 ± 0.008 ^m^	4.93 ± 0.036 ^j^
Nutrivant	6.26 ± 0.052 ^b^	6.42 ± 0.037 ^a^
Peters 2	4.80 ± 0.017 ^k^	5.37 ± 0.010 ^g^
UMAX	5.82 ± 0.028 ^d^	6.06 ± 0.024 ^c^
G2	4.73 ± 0.006 ^kl^	5.24 ± 0.049 ^h^
Yara	6.02 ± 0.045 ^c^	6.27 ± 0.013 ^b^

Mean value ± standard error of three replicates, ^a–m^ represent the significant difference based on Tukey’s HSD test.

**Table 2:** Effect of various fertilizer combinations (different NPK ratios) on number of clusters per plant recorded during experimental years 2017 and 2018.

Fertilizer Source	Clusters per Plant		Mean
	2017	2018	
CK	13.77 NS	19.433	16.6 NS
Zhanlan	14.43	15.767	15.1
Garsoni	15.10	16.433	15.767
Acron	15.43	15.767	15.6
Norway	18.77	14.1	16.433
Peters1	14.10	13.767	13.933
Nutrivant	16.10	16.767	16.433
Peters 2	15.77	16.767	16.267
UMAX	12.77	18.1	15.433
G2	16.10	13.1	14.6
Yara	11.10	14.1	12.6
Mean	14.86NS	15.83	

NS represents the non-significant different between different treatments.

**Table 3:** Commercial NPK fertilizers soil applied on three growth stages (initiation and termination of fruit setting and mid of veraison stage) of grape plants.

	Fertilizers Trade Name	N	P	K
1	CK	0	0	0
2	Zhanlan	16	16	16
3	Garsoni	15	15	15
4	Acron	16	15	15
5	Norway	21	7	12
6	Peters 1	30	10	10
7	Nutrivant	14	14	30
8	Peters 2	20	20	20
9	UMAX	15	15	15
10	G2	20	20	20
11	Yara	15	15	15

**Table 4:** List of primer sequences used in qPCR analysis.

	Gene Name	Sequence of Forward (5′-3′)	Sequence of Reverse Primers (5′-3′)
1	*NR*	ACCGACAGCATCCTCATCAA	AGGTGTTGGGAGATGATGCA
2	*GS*	AGAAGATTGACTGGTCGCCA	CTTTTCAGTGTCACGGCCAA
3	*GDH*	CTTTCGGCTTGGCATCAACT	ATACCGGCCACACTTTGTTG
4	*AS*	GAATTGCACTTCATTGGCGC	GCTGTCACTGCCAACATGAA
5	*HKT1*	GGTTCCTACTGTTCTGGGCT	CGCCTGTATGCCTTGAGTTC
6	*NIR*	ATGCCTGCCACAAAGAAAGG	ACAAACTGGGAGGACATCGT
7	*PHT1–4*	GTACTCTTGCCGGACAGCTC	GCCAGAACCTGAAGAAGCAC
8	*PHT2–1*	CAAGGCTTTAGCCTGTGGAG	CATGGGCAAATGACATGAAG
9	*PHO-1*	ACCCAAGTTGACGCCATT	GAGCAGACTATGTTGCGAATG
10	*PAP*	ACCTGATTATTCTGCTTTCCG	TCCTGCTTCCCAGCTTACTT
11	*SIRK*	GCTTTCAGCCTCCTGTTCAC	CACCAATCCAGGTTCGTTCT
12	*SORK*	AGGAAGCAACGAAGACGAAA	CACGAGCCTGCATAGTTCAA
13	*KUP1*	TGAGCTTTGAAACATGGGAAGACT	TTCTTGTTACCAAGCCTTCCGG
14	*KUP2*	ATGCTTCCTGCCATTTCCACATA	GGTTGGCATGGTTTATATCGTCTG
15	*PAL*	GCCAATCCTGTCACCAACCAT	TGATGGCAGTCCATTGTTGT
16	*CHS*	GTTCTGTTTGGATTTGGACCAG	GTGAGTCGATTGTGTAGCAAGG
17	*F3′H*	GCCTCCGTTGCTGCTCAGTT	GAGAAGAGGTGGACGGAGCAAATC
18	*F3′5* *′* *H*	AAACCGCTCAGACCAAAACC	ACTAAGCCACAGGAAACTAA
19	*DFR*	GAAACCTGTAGATGGCAGGA	GGCCAAATCAAACTACCAGA
20	*LDOX*	AGGGAAGGGAAAACAAGTAG	ACTCTTTGGGGATTGACTGG
21	*UFGT*	GGGATGGTAATGGCTGTGG	ACATGGGTGGAGAGTGAGTT
22	*OMT*	CTCTGCAGGCGCCTCTATTA	CCCAAAACAGAGTCTGGACA
23	*GST*	ACTTGGTGAAGGAAGCTGGA	TTGGAAAGGTGCATACATGG
24	*UBIQUITIN1*	TCTGAGGCTTCGTGGTGGTA	AGGCGTGCATAACATTTGCG

## Data Availability

Data is contained in the manuscript.
